# Skin in the Game: Implementation of Weekly Active Surveillance in a Pediatric ICU

**DOI:** 10.1097/pq9.0000000000000701

**Published:** 2024-02-21

**Authors:** Erica Eberhard, Grace Brooks, Julie LeBlanc, Natalie Lu

**Affiliations:** 1 From the Children's Minnesota, Minneapolis, MN.

## INTRODUCTION

When stratifying pressure injury data by unit, most hospital-acquired pressure injuries (HAPI) at our organization occur in the Minneapolis campus pediatric intensive care unit (PICU-M). The Cardiovascular Intensive Care Unit implemented weekly surveillance in 2020 and saw success with reduced HAPI. In 2022, with engagement and support from leadership, we spread this successful harm reduction tactic to our PICU-M.

## OBJECTIVES

Children’s Minnesota is on a Journey to Zero (JTZ) with a global aim to eliminate preventable harm. A 20% reduction goal was set to decrease the HAPI rate in 2022 compared with 2021.

## METHODS

PICU patient care leaders collaborated with patient safety and wound ostomy care APRNs to establish weekly HAPI active surveillance. Clinical nurses interested in HAPI prevention were identified to fill a JTZ Champion role and trained to recognize potential HAPI, perform pressure injury prevention bundle observations, and provide peer coaching on best practices while promoting psychological safety. Patient care leaders scheduled JTZ Champions one day per week to assess critically ill patients on the unit for HAPI.

## RESULTS

**Figure 1.**
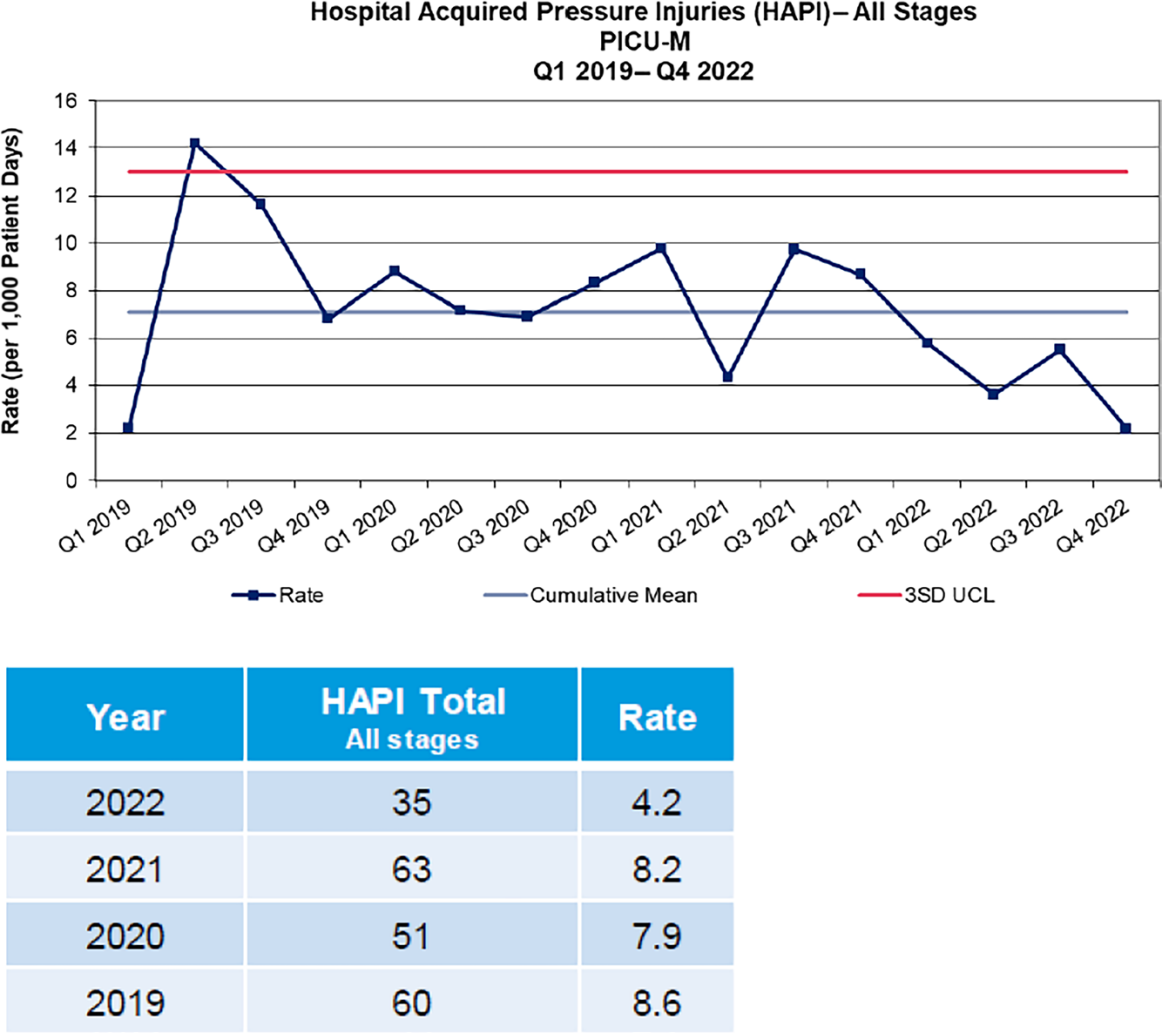
Demonstrates the rate of pressure injury occurrence over 16 quarters or 4 years and the decrease in occurrence with implementation of active surveillance.

**Figure 2.**
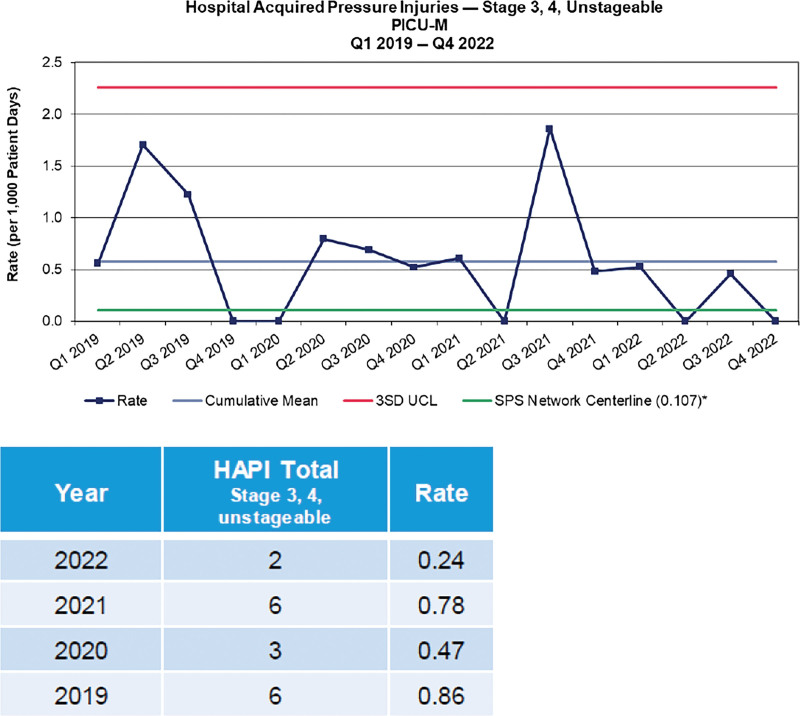
Demonstrates the rate of state reportable pressure injury occurrence over 16 quarters or 4 years and the decrease in occurrence with implementation of active surveillance.

While implementing weekly active surveillance, the HAPI rate for all stages decreased by 48% in 2022 compared with 2021 (Fig. 1) and 69% for Stage 3, 4, and Unstageable HAPI (Fig. 2).

## CONCLUSIONS

This project demonstrates the successful implementation of existing best practices in reducing harm and improving a culture of safety. Weekly active surveillance helps with the early identification of HAPI and provides an opportunity to mitigate risks and perform safety coaching on pressure injury prevention. The weekly cadence increased awareness of HAPI prevention among clinical nurses on the unit and paused the flow of care delivery to think critically about best practices. Providing clinical nurses who are closest to the work with safety training gives them the skills, knowledge, and ability to prevent patient harm.

## IMPLICATIONS

Results and conclusions of implementation fuel the recommendation to continue this process in units that have adopted weekly active surveillance and to expand to neonatal intensive care units and other priority healthcare-associated condition prevention work throughout the organization.

